# Targeting the “hallmarks of aging” to slow aging and treat age-related disease: fact or fiction?

**DOI:** 10.1038/s41380-022-01680-x

**Published:** 2022-07-15

**Authors:** Maryam Keshavarz, Kan Xie, Kristina Schaaf, Daniele Bano, Dan Ehninger

**Affiliations:** 1 Translational Biogerontology Lab, German Center for Neurodegenerative Diseases (DZNE), Venusberg-Campus 1/99, 53127 Bonn, Germany; 2 Aging and Neurodegeneration Lab, German Center for Neurodegenerative Diseases (DZNE), Venusberg-Campus 1/99, 53127 Bonn, Germany

**Keywords:** Physiology, Diseases

## Abstract

Aging is a major risk factor for a number of chronic diseases, including neurodegenerative and cerebrovascular disorders. Aging processes have therefore been discussed as potential targets for the development of novel and broadly effective preventatives or therapeutics for age-related diseases, including those affecting the brain. Mechanisms thought to contribute to aging have been summarized under the term the “hallmarks of aging” and include a loss of proteostasis, mitochondrial dysfunction, altered nutrient sensing, telomere attrition, genomic instability, cellular senescence, stem cell exhaustion, epigenetic alterations and altered intercellular communication. We here examine key claims about the “hallmarks of aging”. Our analysis reveals important weaknesses that preclude strong and definitive conclusions concerning a possible role of these processes in shaping organismal aging rate. Significant ambiguity arises from the overreliance on lifespan as a proxy marker for aging, the use of models with unclear relevance for organismal aging, and the use of study designs that do not allow to properly estimate intervention effects on aging rate. We also discuss future research directions that should be taken to clarify if and to what extent putative aging regulators do in fact interact with aging. These include multidimensional analytical frameworks as well as designs that facilitate the proper assessment of intervention effects on aging rate.

## Introduction

Life expectancy increased from ~50 years in the early 1900s to over 80 years at present [1]. Factors contributing to this development may include advances in medical care as well as the creation of cleaner, safer, and healthier environments for people to live in [1]. Although this represents a great achievement for human societies, the growth in both the size and the proportion of the elderly population also comes with critical challenges. Advanced age is the main risk factor for many common diseases, such as cancers, cardiovascular disorders, and neurodegeneration [1]. Age-related neurodegenerative diseases, including Alzheimer’s disease (AD), Parkinson’s disease (PD), amyotrophic lateral sclerosis (ALS), and others [2–4], severely compromise the quality of life of affected individuals. Moreover, current demographic developments have substantial socioeconomic implications for health and care systems [5, 6]. Available treatments are symptomatic, despite intensive efforts to develop disease-modifying therapies for these devastating conditions [5, 6].

Among known risk factors for neurodegenerative diseases, the aging process itself has the highest impact [7]. For instance, it has been estimated that the risk for developing AD doubles every 5 years over the age of 65; the risk of death due to AD increases 700-fold between the ages 55 and 85 [8, 9]. Hence, strong mechanistic links between brain aging and neurodegenerative disease have been considered [10] and treatments with putative anti-aging drugs (e.g., rapamycin) have been proposed for clinical trials targeting AD [9]. Thus, studying aging and understanding how exactly aging increases the risk to develop neurodegenerative diseases can provide important clues to inform new strategies for early detection, prevention, and treatment.

The critical outstanding question is: Can aging processes be slowed down? Evidence in nature suggests a positive answer to this fundamental question. For instance, similar pathobiological changes associated with aging develop over very different time scales in different mammalian species [11]. While it may take 70 years for a senile cataract to develop in a human, similar age-related changes develop in horses within 20 years, in dogs within 10 years, and in mice in even only 2 years. Analogous considerations also apply to many other age-related alterations (hair greying, muscle loss, etc.). Although the biology underlying these differences in aging rate are not well understood, these examples demonstrate that similar aging phenomena in comparable tissues can develop over very different absolute time scales. Therefore, there seems to be some plasticity that could be harnessed, in theory, for slowing down the aging process.

Much of what is currently thought to be known about the biological underpinnings of the aging process has been presented in concepts like the “hallmarks of aging” [12–14] which summarize processes claimed to be involved in driving organismal aging phenomena. Here, we carefully examine the evidence presented in favor of such links between these processes and aging. As we will explain in detail below, we identify limitations that are often grounded in the choice of models and/or the way aging is measured. We conclude by outlining experimental designs that are suited to overcome these current limitations and that can be used to address if and to what extent putative aging regulators are in fact involved in regulating organismal aging rate.

## The “hallmarks of aging”

The aging field has grown significantly during the 2000s, leading to the testing of many previous hypotheses as well as the emergence of new ideas in the field [15]. In 2013, López-Otín and colleagues published a paper in which they proposed nine hallmarks as the main causes of aging: genomic instability, telomere attrition, epigenetic alterations, loss of proteostasis, deregulated nutrient sensing, mitochondrial dysfunction, cellular senescence, stem cell exhaustion, and altered intercellular communication [13]. This paper soon became a major reference for researchers in the aging field and beyond, accumulating over 1000 citations per year in recent years [15]. The “hallmarks of aging” also inspired many scientists from other fields, including the field of neurodegeneration, to shape their findings in the form of these nine hallmarks [7, 16]. In fact, researchers from different fields ranging from evolutionary biology [17, 18] to biomedical research [1, 7, 19–23] take these nine hallmarks as a base for their research and tend to think of their findings as being relevant to aging if they can identify compatibility with any of these nine hallmarks. While this clearly shows the importance of aging research for an understanding of different biological and medical issues, a key question here is to what extent these nine hallmarks of aging represent the “causes of aging”. In other words, on which foundational evidence and assumptions have the “hallmarks of aging” been built? To address this question, we have performed a systematic analysis of the papers that were used as supporting evidence for the involvement of each of these hallmarks in the aging process and identified important limitations which need to be discussed and acted upon.

### Lifespan —a valid proxy for aging?

Lifespan is often used as a proxy marker for aging. That is, interventions (e.g., genetic manipulations, pharmacological treatments, or other environmental interventions, such as manipulation of dietary factors) found to extend lifespan in model organisms (such as in mice, flies, or worms) are concluded to slow aging because they extend lifespan.

**Fig. 1:**
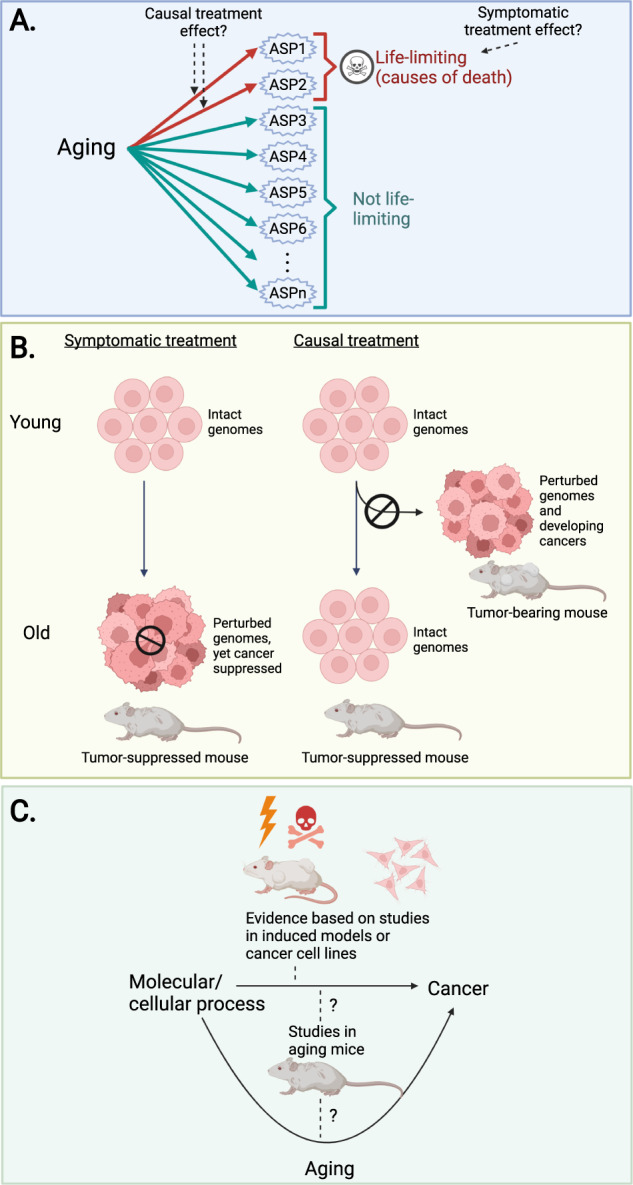
Lifespan is not a sufficient readout of aging. **A** Only a subset of aging-associated changes (shown here as a collection of age-sensitive phenotypes; ASPs) is life-limiting in the context of natural aging. ASPs could exert their effects on lifespan either individually or via combinatorial effects. Experimental lifespan extension implies that the given intervention interacts with the life-limiting subset of ASPs, but does not speak to the intervention’s ability to influence any of the ASPs that are not life-limiting. In addition, treatments that extend lifespan could be targeting the cause of reduced lifespan in a symptomatic fashion (e.g., inhibition of cancer growth using a cytostatic drug), rather than tackling its underlying causes (e.g., inhibition of mutagenesis to prevent cancer formation in the first place). For further discussion, see the main text. **B** Symptomatic and causal treatments can lead to the same outcome, but through different mechanisms. The differences between symptomatic and causal treatment are shown here using the age-related pathology “cancer” as an example. Under symptomatic treatment, tumor growth is blocked by non-specifically inhibiting cell proliferation via a cytostatic drug. Importantly, however, the age-related accumulation of genome damage (that underlies cancer predisposition in old age in our example) remains unaffected by this type of approach. Causal treatment prevents the aging-associated accumulation of genome damage, thereby inhibiting cancer by targeting the biology underlying the age-related increase in cancer predisposition. **C** Treatment-induced anti-cancer effects in aged mice could, in principle, be explained by either anti-aging effects (treatment targets aging-associated changes that predispose for cancer formation in old age, such as genomic instability), by direct anti-cancer effects (i.e., age-independent inhibitory effects on neoplastic diseases, such as a general inhibition of cell division) or by a combination of these two effects. However, direct anti-cancer effects are sufficient to explain any observed anti-cancer effects if a treatment exerts such effects in experimental contexts that do not involve aging, such as experiments performed in young mice (e.g., with chemically induced tumors) or in cell culture models.

The problem with this assertion is that natural lifespan is often restricted by specific sets of aging-associated pathologies, not by some sort of generalized physiological decline. As a consequence, lifespan-extending interventions are likely to exert their effects on lifespan by targeting whatever pathology is life-limiting in the context of natural aging in that species (hereafter termed lethal age-sensitive phenotypes; lethal ASPs; these might be lethal in isolation or become lethal via combinatorial effects). In species in which lifespan is limited by a very narrow set of pathologies (e.g., specific cancers), treatment-induced longevity effects indicate that this intervention has an effect on the small set of lethal ASPs in this species (Fig. 1A). It is important to note, however, that the observation of such treatment effects has no implications concerning any of the potentially much larger subset of age-dependent changes that do not limit lifespan per se (non-lethal ASPs; such as hair greying, skin aging, sarcopenia, osteoporosis, cognitive decline, etc.; see Fig. 1A).

Also, note that treatment-induced longevity effects do not necessarily imply that treatment targets the processes causally underlying the aging-associated development of lethal ASPs. While this is one possibility, pro-longevity effects could also be caused by symptomatic treatment effects on lethal ASPs. For instance, a cytostatic drug may extend lifespan by (symptomatically) inhibiting lethal neoplastic disorders; it would do so by generally inhibiting cell proliferation [24] and not by influencing the mechanisms favoring the development of lethal neoplastic disorders in old age (e.g., genomic instability and mutation accumulation in the context of aging) [25–27].

The “hallmarks of aging” paper [13] makes extensive use of data derived from lifespan studies to support claims about roles in the general biology of aging. To make this point clear, we identified all the references cited within [13] that studied genetic variants, dietary factors or pharmacological treatments and showed lifespan extension in the context of natural aging in any one of the different model organisms used (Supplementary Table 1 and Supplementary Table 2). The results are discussed below separately for each species, *M. musculus*, *D. melanogaster*, and *C. elegans*.

Across a range of mouse strains, cancers have been shown to account for ~70 – 90% of natural age-related deaths [28–32]. Hence, given that cancer is the main known life-limited pathology in mice, any pro-longevity intervention in this species is likely exerting its effects via inhibition of carcinogenesis. Again, to examine this point in greater detail, we extracted all genetic, dietary and pharmacological pro-longevity interventions that were cited in [13] and analyzed whether or not each of these interventions has been previously demonstrated to have anti-cancer effects. Consistent with the statements above, these analyses revealed links to cancer inhibition in >80% of these interventions (Table 1) [32–79], indicating that anti-cancer effects could in fact largely explain the lifespan extension induced by these pro-longevity interventions (i.e., there is no need to assume that general aging-associated physiological decline was slowed to explain the pro-longevity effects). Cancer-inhibitory effects were due to a range of mechanisms, including the suppression of de novo cancer formation and the inhibitions of established tumors by reducing cancer growth, promoting apoptosis, and/or inhibiting angiogenesis [32–79].

**Table 1:** Established anti-cancer roles of the “hallmarks of aging”.

The table shows interventions (experimental conditions; examined in mice in the context of natural aging) presented in the “hallmarks of aging” paper [13] as evidence to support a role of each of the given “hallmarks” in aging. For each intervention, the table indicates whether the intervention is associated with known anti-cancer effects or not (in either aged mice and/or cell lines/“induced” mouse models). Note that cancer-inhibitory effects have been established in most cases, indicating that pro-longevity effects of these interventions can be explained by suppressing lethal neoplastic disease in the context of aging.

As discussed above, pro-longevity interventions in mice could, in principle, extend lifespan by targeting the root causes of aging-associated cancers (e.g., by promoting genomic stability / inhibiting mutagenic events). Alternatively, they could inhibit cancers in symptomatic ways, i.e., by tapping into mechanisms unrelated to the ones that mechanistically link aging and cancer formation (this would be the case, for instance, in the scenario where a cytostatic drug inhibits general cell proliferation but leaves genomic instability/mutation accumulation unaffected, or even enhances it due to mutagenic properties that can be associated with this class of drugs [80]). Note that both of these scenarios could cause aged animals to have a lower cancer burden (Fig. 1B). However, it would not be meaningful to consider the animals treated with the symptomatic treatment (the cytostatic drug) to benefit from “slowed aging”. After all, they are as cancer-prone as untreated age-matched controls (or even more cancer-prone considering mutagenic properties of cytostatic drugs) (Fig. 1B) and this would become evident as soon as the symptomatic treatment is stopped. Animals treated with a causal treatment, in contrast, would show a causally reduced cancer risk (due to preserved genomic integrity) (Fig. 1B) that persists even if treatment is terminated.

If a treatment’s anti-cancer effects have been established in the context of naturally aging mice, it is not possible to distinguish between these two scenarios; anti-cancer effects could be due to either symptomatic or causal intervention effects. A distinction between these scenarios is, however, possible in cases in which experimental designs allow the dissociation of direct anti-cancer effects from anti-cancer effects that could be indirectly caused by anti-aging effects. This is possible whenever experiments are carried out in “non-aging” contexts, such as in chemically-induced cancer models (established in young mice) or in cell culture models of cancer (Fig. 1C).

Again, when the manipulation of putative aging regulators (Table 1) suppresses cancers in induced mouse cancer models (in which cancers are induced either chemically, genetically, by radiation, or via xenograft models [81–83]), this strongly implies a direct and symptomatic effect (Fig. 1C). In these mouse models, cancers are generated in young mice and therefore no role of anti-aging effects can be attributed to the anti-cancer effects in these contexts. Similarly, anti-cancer effects in cancer cell lines are also an indication for symptomatic effect (Fig. 1C). Table 1 summarizes the different types of anti-cancer evidence for each of the putative aging regulators considered here. Interestingly, for 100% of these interventions, an anti-cancer role has been shown in “induced” mouse cancer models. For 93% of these interventions, an anti-cancer effect has also been found in cancer cell lines. These observations strongly support the notion of direct and aging-independent anti-cancer effects. As a consequence, given that aging-independent anti-cancer effects are documented for most of the putative aging regulators (Table 1), a straightforward, yet underappreciated mechanistic explanation for much of the pro-longevity effects afforded by these interventions in mice is that they are not induced by “slowing aging” but rather by the direct inhibition of lethal neoplastic disease via aging-independent mechanisms. These considerations point to serious flaws in the sole use of longevity as a proxy marker for aging studies in mice.

While major causes of age-related death in mice have been identified (see above), processes limiting lifespan in *Drosophila melanogaster* are not that well understood [84, 85]. In flies, the intestinal epithelium constitutes an important barrier against microorganisms and environmental toxins [84]. The structure and function of the intestinal epithelium significantly decline in aging flies, to a point that is thought to become life-limiting [84, 86–88]. One of the well-described pathologies in the aged fly intestine is epithelial dysplasia, driven by intestinal stem cell (ISC) over-proliferation which leads to an increase in intestinal progenitor cells and aberrant differentiation [89–91]. Interestingly, several well-known pro-longevity interventions in flies, such as the mTOR inhibitor rapamycin, caloric restriction, and genetic loss-of-function mutations targeting insulin/insulin-like growth factor signaling (IIS), have been shown to slow down the proliferation rate of ISCs, to delay intestinal dysplasia and to extend lifespan [86, 91–94]. More importantly, several studies have shown that genetic manipulations specifically targeted to ISCs are sufficient to extend lifespan in flies [86, 95–100] (Table 2), consistent with the notion that intestinal dysplasia is a life-limiting pathology in this species. These observations also demonstrate, for flies, that lifespan extension can be induced by eliminating or reducing only one specific life-limiting ASP (without necessitating broader effects on aging). Intriguingly, *Drosophila* females demonstrate a greater level of intestinal dysplasia compared to males [91] and in line with this, female flies usually also show a greater longevity response to these interventions [101–106].

**Table 2:** Examples of ISCs-specific interventions that extend lifespan in Drosophila.

“Hallmarks of aging”	Intervention	Reference
***Loss of Proteostasis***	PERK downregulation	[95]
	Hsp68 overexpression	[86]
***Deregulated Nutrient-Sensing***	Inhibition of JNK signaling	[86]
	Overexpression of Jafrac1	[86]
	Downregulation of lnR	[86]
	Downregulation of Dp110	[86]
	Downregulation of Akt	[86]
	Downregulation of dMfn	[96]
	Activation of Drp1	[96]
	Downregulation of Bsk and BskD	[86]
***Mitochondrial Dysfunction***	Overexpression of dPGC-1	[97]
	Overexpression of Ndi1	[98]
***Altered Intercellular Communication***	Activation of Kif1a	[98]
	PGRP-SC2 overexpression	[99]
	Overexpression of Ssk	[100]
***Stem Cell Exhaustion***	Overexpression of dPGC-1	[97]

The table summarizes genetic interventions that have been shown to be sufficient to extend lifespan in *D. melanogaster* when targeted to intestinal stem cells (ISCs).

It has been shown that specific genetic perturbations in the muscle [107, 108], fat body [101, 109], brain [101, 110] and neurosecretory cells [102, 111, 112] also are sufficient to extend life span in *Drosophila melanogaster*, however, the underlying mechanism is poorly understood. Note that ISC proliferation is also regulated via extrinsic cues derived from distant tissues, such as muscle, brain, trachea, and fat body [113–116]. In line with this, it has been shown that inhibition of neuronal Hh results in an increase in the number of intestinal progenitor cells as well as defective differentiation, whereas activation of neuronal Hh signaling reduces intestinal progenitor cells, significantly improves intestinal homeostasis [115], and leads to lifespan extension in *Drosophila melanogaster* [110]. More research is needed to investigate the existence of other possible life-limiting pathologies in other tissues which could be the cause of age-related death in flies.

The roundworm *Caenorhabditis elegans* is another important model organism for aging research and a large number of genetic and environmental factors have been identified to extend its lifespan. However, what specific life-limiting pathologies naturally play a role in *C. elegans* remains to be better understood. A longitudinal analysis in *C. elegans* showed that pharyngeal pumping span (the length of the time interval the pharynx is active) is positively correlated with adult lifespan [117]. Recently, another study revealed that pharyngeal infections and deterioration are among the main life-limiting pathologies in worms [118]. In this study [118], two types of deaths have been reported in adult *C. elegans*: an early death with a swollen, infected pharynx and a later death with pharyngeal atrophy [118]. Interestingly, additional work analyzing some of the well-known long-lived *C. elegans* mutants, such as *glp-1*, *eat-2*, *ced-1*, and *daf-2* mutant lines, revealed that these interventions change the frequency and/or timing of either form of death, thereby leading to an increase in lifespan [118–120]. Again, these observations in *C. elegans* are also in line with the notion that the rescue of one specific life-limiting pathology may be sufficient to explain lifespan extension. However, more work is needed to further define life-limiting changes in *C. elegans* and to explore how those are affected in longevity mutants.

Together, the data and considerations discussed thus far indicate that isolated pro-longevity effects of an intervention, without any further studies, are insufficient to support strong conclusions about any possible broader anti-aging effects this intervention may have.

### Choice of models

#### Shortened lifespan

Conclusions about the biology of aging have also been drawn on the basis of lifespan studies identifying factors that *shorten* lifespan. As shown in Supplementary Table 1, work focused on shortened lifespan is used to back up claims about aging in [13].

The assertion that lifespan shorting effects can be used to inform the biology of aging is problematic because lifespan can be shortened in many ways entirely unrelated to factors that naturally limit lifespan during aging (lethal ASPs) or are otherwise associated with aging-related phenomena (non-lethal ASPs). There are numerous examples of animal models that live shorter for reasons completely unrelated to aging. For instance, mutations in the *TSC1* or *TSC2* genes cause tuberous sclerosis complex (TSC) in humans, a neurogenetic condition associated with autism spectrum disorder, epilepsy, and intellectual disability [121, 122]. Neuron-specific conditional *Tsc1* and *Tsc2* mouse mutants exhibited early premature death due to severe brain pathology [123, 124]. Treatment with an mTORC1 inhibitor rescued brain pathology in the mutant mice and resulted in an increase of lifespan [123]. This is a clear example where a mutation shortens lifespan, yet the cause of death is in no way related to aging and also the mTORC1 inhibitor rescue effects are not related to any aspect of the aging process.

#### Models that aim to phenocopy aging

Inferences about aging have been also drawn from studies analyzing models that are claimed to phenocopy manifestations of aging but in which the link to aging is either weak or unclear. This includes, for instance, mitochondrial DNA (mtDNA) mutator mice, irradiated mice, mice featuring diet-induced obesity, telomere-dysfunctional mice, mouse models with persistent expression of *Wnt1*, mouse models with conditional deletion of *Tsc1* or mouse models of rare genetic syndromes termed progerias (Werner syndrome (WS), Hutchinson-Gilford syndrome (HGPS)). The latter feature a premature manifestation of some phenotypic changes reminiscent of those observed during normal aging. It remains unclear, however, how these conditions relate to aging. While WS patients, for instance, show a premature manifestation of some phenotypic changes seen in elderly people, such as greying and loss of hair and a development of cataracts, they do not develop prematurely many other consequences of aging, such as cognitive decline, immune dysfunction or cardiovascular disease [125, 126]. In addition, the epithelial and hematopoietic tumors which are very commonly seen during normal aging in humans, are not more common in WS patients [125, 126]. Instead, they show mesenchymal tumors which are rarely seen in normal elderly people [126]. Hence, the relevance of these genetic conditions as models for aging is unclear and it is debated whether they can provide valuable clues about processes involved in aging [126].

As shown in Supplementary Table 1, work focused on models that aim to phenocopy manifestations of aging is used very frequently in [13] to support claims about aging, in particular with regards to some specific “hallmarks”: Approximately 67% of studies cited to support a role for genomic instability in aging were based on such models; also, 63% cited for cellular senescence and 62% cited for stem cell exhaustion refer to studies that were actually not focused on normal aging but instead on models with unclear relevance to aging, such as the ones outlined above.

#### Cell culture models/Cell-centric view on aging

**Fig. 2:**
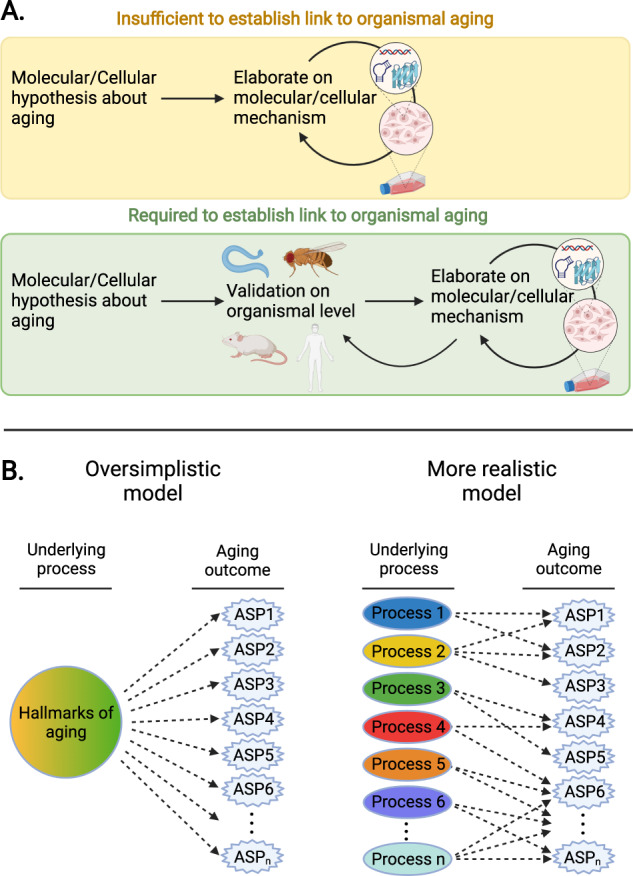
Organismal aging needs to be studied in organismal models. **A** Examining molecular and cellular hypotheses about aging on the cellular level (cultured cells) is insufficient to truly establish a link to aging. Instead, hypotheses about aging must be validated at the organismal level before potentially additional molecular and cellular studies can be used to elaborate on mechanisms (provided suitable model systems are available). **B** Aging is a series of outcomes with diverse contributing factors. Rather than a set of outcomes arising from a small set of processes (“hallmarks of aging”), aging outcomes are likely shaped in complex ways by a large number of factors. *ASP* age-sensitive phenotype.

Organismal aging cannot be simply mimicked in a cell culture dish because it is an emergent property of intact organisms. Hence, examining molecular and cellular hypotheses about aging on the cellular level (cultured cells) does not necessarily yield insights that are relevant to the aging process on the organismal level. If an intervention improves or accelerates cellular function in cultured cells, this observation has, in isolation and without further organismal-level validation in an aging context, no implications regarding organismal aging (Fig. 2A).

For instance, reactive oxygen species (ROS) have been hypothesized for a long time to play a role in driving organismal aging. While cells and organisms deficient in ROS defense mechanisms are in fact exquisitely sensitive to oxidative stressors [127–129], only in vivo experiments in the context of natural aging were able to address the question whether and to what extent ROS play a role in organismal aging. However, such studies showed that increased ROS not only does not accelerate aging, but did even extend lifespan in yeast and *C. elegans* [130–132]. Analyses in mice also showed that genetic manipulations that increase mitochondrial ROS and oxidative damage do not accelerate aging [133, 134] and that manipulations that increase antioxidant defense did not extend longevity [135]. This is a clear example illustrating that cellular damage models require validation in in vivo aging models if they are intended to deliver insights about aging.

Telomere attrition, which is claimed by some to cause organismal aging [13, 136, 137], represents another example of cell-centered models that have been extrapolated to organismal aging despite a lack of evidence on this level of analysis. The relationship between telomere attrition and cellular senescence in vitro has fueled claims that telomere length is a determinant of organismal aging and lifespan [138, 139]. There have been numerous studies on the importance of telomere length on replicative cellular lifespan in human cultured cells [140–143]; however, studies on how telomere attrition could be involved in natural organismal aging are indeed very limited and controversial. Analyses of several different mouse strains revealed no significant correlation between telomere length and longevity in closely related mouse strains and mice with naturally relatively shorter telomere lengths show no significant reduction in lifespan [144]. However, in general, mice have much longer telomeres than humans [145]. As a consequence, mice do not seem to show functionally relevant telomere attrition that takes place during their normal lifespan, indicating that telomere attrition may in fact not underlie aging phenomena observed in these wildtype stocks of mice. Mice engineered to develop short telomeres (Terc-deficient mice) do not show significant adverse effects on many health parameters (lifespan, motor behavior/activity, histological measures, weight gain, etc.) in the first generation. A lifespan-shortening effect becomes only evident after multiple rounds of breeding [137, 146], suggesting that only progressive telomere attrition accumulating across multi-generational cell divisions is capable of eliciting effects on lifespan and health-related outcomes in these mouse models. Such extreme telomere attrition cannot be seen within a normal mouse life [147]. Furthermore, even after multiple generations, these mice feature a pattern and spectrum of pathologies (skin ulceration, infertility, increased frequencies of very specific forms of neoplasias, and frequently lethal gastrointestinal lesions) that looks very different from the organismal changes taking place during normal aging in both mice and humans [126, 137, 146]. Moreover, many studies (with a few exceptions [148, 149]) showed that increasing telomere length promotes carcinogenesis, whereas telomerase-deficiency (leading to telomere attrition) suppresses cancer formation in a number of murine cancer models [150–155]. Studies in other animal models (beyond the mouse), such as in zebrafish (*Danio rerio*), *D. melanogaster* and *C. elegans*, as well as studies in plants (*Arabidobsis thaliana*) also question the causal involvement of telomeres in aging (see this review for a more detailed discussion [147]). Hence, to date, there is insufficient evidence to support strong conclusions about telomere attrition playing an important role in organismal aging.

Advances in stem cell biology have led some to speculate that brain organoids produced from human pluripotent stem cells (HPSC) could be utilized as a replacement for in-vivo studies. While brain organoids provide an intriguing environment for studying complicated cell-cell interactions, modeling age-related neurodegenerative disorders remains difficult. Brain organoids have a transcriptional profile similar to that of the prenatal brain, and, while they may be suitable to model some features of brain development, their relevance as models for aging-associated change is less clear [156]. Another disadvantage is the lack of complete vascularization, which precludes the modelling of key aspects of brain physiology [157]. Brain organoids also lack the full cellular diversity present in the mammalian brain. Several brain organoids systems, for example, contain astrocytes but no microglial cells [158]. Some others consist of neurons and glial cells but not oligodendrocytes [159]. Furthermore, a wide range of differentiation techniques results in variability in size and structure of brain organoids, hence presenting challenges for reproducibility of research [160, 161]. As a consequence, brain organoids are at present still basic and immature and will require much more additional development.

Altogether, we conclude that links between a molecular/cellular process and aging have to be established in the context of organismal aging to support claims about roles of that process in aging (Fig. 2A). This is because organismal aging is a property emerging in whole intact organisms as time passes by. As such, reductionist systems, like cell culture models, may not necessarily capture processes and features relevant to organismal aging.

### Underestimating the complexity of the aging process

Aging is the process that progressively transforms young adult organisms into aged ones with changes across multiple physiological systems [11, 162–165], leading to the emergence of a large number of age-sensitive phenotypes (ASPs), such as loss of bone density, skin thinning, atherosclerotic changes, reduced kidney function, etc. [164]. One of the implicit assumptions in [13] is that putative aging regulators are involved in the development of the majority (if not all) ASPs. However, the complexity of biological systems suggests this is likely an over-simplistic view (Fig. 2B). Recent multi-omics research indicates that aging-associated change take place at varying rates in different organs and systems; also, the underlying biological mechanisms might differ across tissues [166, 167]. It has also been shown that, while some downstream processes are conserved across tissues, transcription factor regulatory networks have limited overlap [166]. In general, if a given intervention improves or accelerates one or a few ASPs in a given organism, this observation cannot be simply extrapolated to other ASPs or organisms. However, such an extrapolation is commonly seen, for instance, when interventions are concluded to slow aging based on the assessment of a small number of ASPs (Supplementary Table 1). As we will discuss in more detail below, considering only a small number of ASPs is insufficient to draw strong conclusions about broader effects on aging.

### How can we measure aging: Large-scale assessment of aging outcomes

Aging is a multifactorial process that occurs as adult organisms mature into aged organisms. The results of aging are widespread functional changes across virtually all organ and tissue systems, an increased risk to develop age-related diseases, as well as an elevated mortality risk [126, 162–164]. The process of aging occurs gradually, with changes manifesting across almost all tissue and organ systems and across all levels of biological complexity (i.e., molecular, cellular, tissue and organismal levels of analysis) [164, 168].

**Fig. 3:**
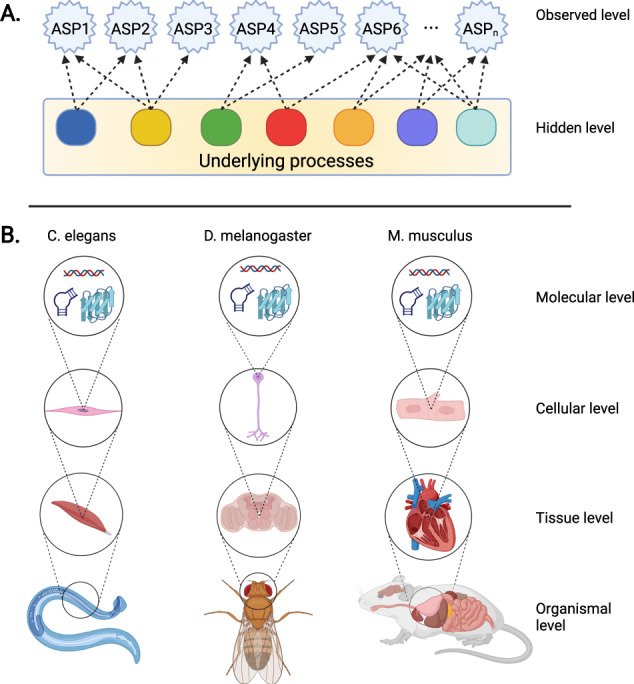
How to measure aging. **A** Aging is a collection of processes with well-characterized and observable phenotypic outcomes (age-sensitive phenotypes; ASPs) but often poorly understood underlying causes. It is likely that a large number of underlying processes drive age-dependent phenotypic changes, each contributing to some of the observed phenotypes. ASPs can be measured directly and can be used as markers to address whether a given intervention (e.g., genetic, dietary or pharmacological) targets the processes underlying their age-dependent change (even if the processes themselves remain poorly defined). **B** ASPs can be measured in many organisms and across levels of biological complexity (for example, see main text).

Although the mechanistic processes of aging are not well understood and are therefore difficult to quantify, it is straightforward to measure “outcomes” of the aging process (Fig. 3A). In other words, aging is a process with a variety of clearly observable outcomes, such as loss of muscle mass, a loss of bone density, increased numbers of skin wrinkles, hair greying, low-grade tissue inflammation, etc. which can be measured even in the absence of knowledge of their underlying causal processes (Fig. 3A). A subset of parameters may be amenable to longitudinal assessment over time (to derive within-subjects rate of change estimates), others may require cross-sectional study designs comparing young adult vs. aged animals (e.g., all parameters linked to data collection in the context of terminal procedures; these can be used to derive population-level rate of change estimates).

An example of this approach are deep-phenotyping studies, which have been used to analyze a wide range of aging-related phenotypic changes across various tissues and organ systems, providing a multi-dimensional view on the phenotypic consequences associated with aging [32, 36, 169–171]. Among hundreds of parameters examined in young and old animals, many differed between the young and old animals, identifying them as ASPs. Once ASPs are identified for any given organism, they can be used collectively as a multi-dimensional representation of aging-associated phenotypic change, which in turn can be used to test putative anti-aging interventions (PAAIs) against. This approach has been successfully used to determine whether PAAIs, such as pharmacological mTOR inhibition using rapamycin, food restriction employing an every-other-day feeding regime or genetically inhibiting growth hormone signaling in dwarf mutant mice, indeed delay aging [32, 36, 169–171].

Remarkable technological advances facilitate an ever-increasing ability to create large-scale phenotypic maps of aging-associated change in model organisms (Fig. 3B), spanning from the molecular to the organismal level. For instance, the assessment of age-dependent phenotypic change in mice can include, but is by no means limited to, an examination of behavioral and neuropsychiatric functions (e.g., learning and memory, attention, sensorimotor gating, motor functions, sensory functions) as well as neuroanatomical (e.g., MRI, histopathology) and neurochemical (e.g., neurotransmitter analyses) measurements, an assessment of endocrine functions (e.g., plasma hormone concentrations, histopathological changes in the thyroid gland, adrenal gland etc.) and metabolism (e.g., body composition changes assessed by NMR, changes in energy metabolism assessed via indirect calorimetry, glucose tolerance measurements, surface and core body temperature measurements, food and water intake, analyses of substrate turnover rates), as well as structural and functional analyses focused on the cardiovascular system (e.g., echocardiography, electrocardiography, blood pressure measurements, histopathological analyses), the respiratory system (e.g., whole body plethysmography, histopathological analyses of lungs and bronchial system), the gastrointestinal tract, liver and pancreas (e.g., histopathological analyses, clinical chemistry, microbiome analysis), the renal system (e.g., assessment of glomerular filtration rate, histopathological analyses, clinical chemistry), skeletal system (e.g., bone densitometry, histopathological analyses), reproductive system (e.g., histopathological analyses, plasma hormone concentrations), immune system (e.g., flow cytometry, antibody measurements, histopathological analyses, immune activation assays), the hematopoietic system (e.g., blood cell counts, histopathological analyses) and the skin (histopathological analyses, transepidermal water loss) [32, 36, 169].

Deep phenotyping approaches are not limited to mammalian model systems. Studies in *Drosophila melanogaster*, for instance, can also draw from a rich set of aging-associated phenotypic changes, which can be utilized collectively as a proxy to measure aging. This includes, but is not limited to, analyses of molecular alterations (e.g., bulk changes in transcriptome [172–175], proteome [176, 177] and metabolome [178, 179]; single-cell transcriptomic changes [180]); neuromorphological changes (e.g., neurodegeneration [181]), behavioral assessments (e.g., learning and memory [182–184], locomotor activity [185, 186], circadian rhythm and sleep patterns [187, 188]), an assessment of muscle structure and function (e.g., changes in muscle morphology and integrity [189]), analyses of changes in heart function (e.g., assessment of cardiac performance [190, 191]) and gut homeostasis (e.g., histopathological analyses of epithelial dysplasia and barrier function [91]).

A multitude of age-dependent changes can also be observed in the roundworm *Caenorhabditis elegans*, another popular model organism in aging research. This includes changes at the molecular level (e.g., changes in transcriptome [192–194] and proteome [195, 196]), the subcellular level (e.g., structural and functional changes in mitochondria [197]), and tissue-specific changes, such as those affecting the reproductive system (e.g., rate of reproduction and progeny number [198, 199], deterioration of germline cells and changes in oocyte morphology [200]), muscles (e.g., assessment of muscle structural integrity and sarcomeres structure [201, 202], pharyngeal muscles morphology [203]), neuromuscular functions and the nervous system (e.g., morphology and function of touch receptor neurons [204, 205], neurite sprouting and synapse deterioration [206], locomotion [117, 207, 208], pharyngeal pumping rate [117], learning and memory [209]). For further details, the interested reader is referred to existing review articles, such as [210].

### Accounting for aging-independent effects

Interventions that slow, delay or even stop aging must, by definition, interfere with the transformation of a phenotypically young to a phenotypically aged organism. Therefore, studying potential interventions only in aged mice is not sufficient to conclude that a PAAI interferes with the aging process; instead, studies must be designed to examine the PAAI in both young and old animals (either using longitudinal or cross-sectional study designs). This is required to support valid conclusions about the nature of the interactions between a PAAI and aging processes.

Based on the above considerations, designing studies that distinguish between an intervention targeting age-dependent change and a mimicry of such an effect is rather simple. One needs to (1) generate knowledge of lifetime trajectories of ASPs to determine when age-dependent changes in ASPs are first detectable to then (2) design experiments that include young treated reference groups that are subjected to PAAI before age-dependent changes in ASPs become detectable. This allows investigators to dissociate PAAI effects on ASPs from age-dependent changes in these ASPs.

**Fig. 4:**
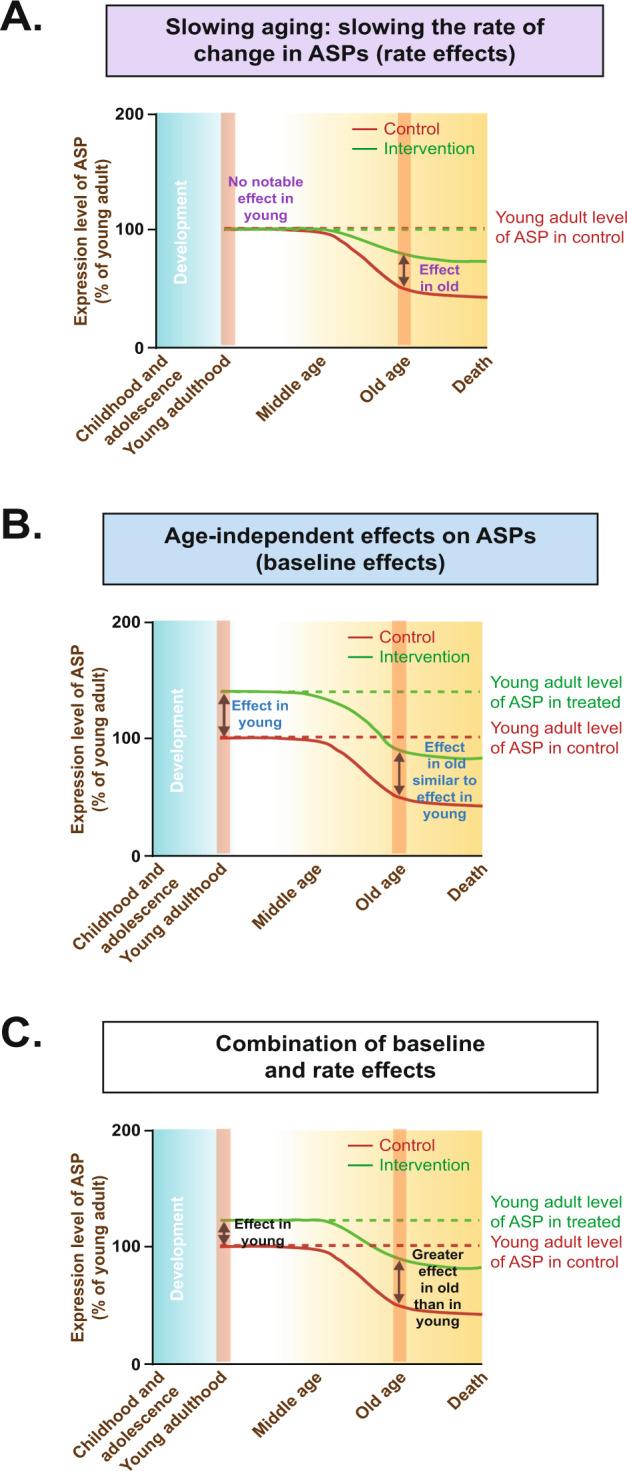
Mechanisms by which putative anti-aging interventions (PAAIs) could influence age-sensitive phenotypes (ASPs). In principle, PAAI effects on ASPs could be attributed to one of the three models: (1) rate model, (2) baseline model, or (3) a combination of rate and baseline models. In the rate model (**A**), a given anti-aging treatment slows the rate at which an ASP develops but does not have any effects on the ASP prior to the manifestation of age-dependent change in the ASP. This pattern supports the interpretation that age-dependent phenotypic change (aka aging) has been slowed by treatment. In the baseline model (**B**), a short-term treatment in young animals has similar effects on ASPs as a long-term treatment in aged animals. This pattern indicates age-independent effects unrelated to any influence on the aging process. It is also possible that a treatment influences ASPs in both young and old mice but with larger effects in old mice than in young mice (**C**). This pattern is more difficult to interpret given that it could be caused by a mixture of age-independent effects and effects on aging rate; alternatively, it could also arise from age-independent effects alone, if treatment duration has an influence on treatment effect size (long-term treatment in old mice resulting in larger effects than short-term treatment in young mice).

For instance, the number of neurons in the mouse brain could be increased by a specific genetic variant that promotes neurogenetic processes during development of the animal, but has no impact on the rate of neuron loss during aging. Hence, this variant would affect aspects of development without influencing aging-associated change. Although this genetic variant would cause animals to have a larger number of neurons in old age, this observation cannot be interpreted as slowed aging because the rate of age-dependent change remains unaffected [35]. Hence, the true nature of an intervention can only be understood through the examination of the intervention’s effect on both young and old animals, which allows the distinction between aging-independent effects on ASPs (such as in the example above) and a slowing of age-dependent changes in ASPs (Fig. 4). An example of the latter scenario may be a genetic variant that delays or slows aging-associated neuron loss through neuroprotective mechanisms but leaves unaffected neurogenetic processes during development (Fig. 4).

This is analogous to the distinction between disease-modifying and symptomatic treatments [211–215]. While both kinds of treatments may have value for the patient, only the former approach targets the underlying cause of the disease, while the latter approach focuses on the presented symptoms. For example, a drug that enhances cognitive function in both Alzheimer’s disease (AD) patients and healthy adults could serve as a symptomatic treatment, but it would not reveal any insights specifically related to the causes of AD. Similarly, a drug that enhances cognitive function in *pre-symptomatic* AD patients cannot have these effects by targeting the causes underlying cognitive decline (because cognitive decline has not yet emerged in pre-symptomatic patients) and, hence, will not give insight into the pathogenesis of AD. Only a treatment that alters the rate of cognitive decline in AD can be considered a disease-modifying treatment and can be used to better understand the causes underlying AD-associated cognitive decline.

There is currently a shortage of studies with suitable designs (which requires the inclusion of treated and untreated young groups of animals, as outlined above) to allow a judgement of whether or not PAAIs slow the rate of aging. Two unbiased large-scale phenotyping studies including young groups had been published previously, one focusing on the mTOR inhibitor rapamycin and one analyzing effects of a dietary restriction regime on a large set of ASPs [32, 36]. Both inhibition of mTOR signaling and dietary restriction represent important cornerstones in the aging field with many links to the “hallmarks of aging” (e.g., mTOR signaling has well-established links to proteostasis, nutrient sensing, mitochondrial dysfunction, intercellular communication and cellular senescence). Interestingly, in both studies it was observed that intervention effects that are specific to the aged group of mice (supporting the notion of a slowed aging rate) were rather rare. Age-independent effects on ASPs, in contrast, were common, indicating that many intervention effects were unrelated to a slowing of aging rate. For instance, the dietary restriction study mentioned above [32] analyzed 116 ASPs. Strikingly, out of these 116 ASPs only 7 ASPs were influenced by dietary restriction in a way clearly consistent with a slowing of aging rate. 33 ASPs, in contrast, were countered by dietary restriction in both young and old mice, which stresses the importance of controlling for age-independent treatment effects.

Another set of important interventions for the aging field represent those targeting (inhibiting) growth hormone signaling. No large-scale phenotyping study has been published until recently. For growth hormone signaling, we therefore reviewed all available data obtained from studies involving long-lived dwarf and related mouse lines (Ames dwarf [216], Laron dwarf [217], Snell dwarf [218], growth hormone receptor knock-out [219], growth hormone releasing hormone receptor knock-out [218] and Igf1 heterozygous mice [220]) (Supplementary Table 3). Overall, in all papers analyzed, we identified 61 ASPs examined in both young and old groups of mice (Supplementary Table 3). Notably, 30 out of 61 ASPs countered and tested in both young and old mice were clearly influenced in an age-independent fashion (similar effects in young and old). Furthermore, for 9 additional ASPs assessed in both young and old mice, there was a non-significant trend towards similar effects in young and old animals. This is in line with a recently published large-scale phenotyping analysis of *Ghrhr*^lit/lit^ dwarf mutant mice that found that most ASPs ameliorated by the mutation were influenced in an age-independent fashion with very similar effects in young and old mice [171].

The available data for rapamycin, dietary restriction and growth hormone signaling-related mutants point towards similar conclusions. First, they indicate that only a subset of ASPs countered by rapamycin/dietary restriction/growth hormone signaling-related mutations follows the rate effect model shown in Fig. 4 (indicating slowed aging). Moreover, these analyses show that even for some of the most intensely investigated PAAIs only limited data are available on ASPs and organismal aging. It is therefore far from clear to what extent PAAIs, based on the “hallmarks of aging” have the capability to slow organismal aging rate. More comprehensive studies, based on large-scale approaches and including both young and aged treated animals, are required to further our knowledge regarding possible links between the putative aging regulators and organismal-level aging phenomena.

## Conclusion and perspective

To date, key concepts regarding the biology of aging (such as summarized, e.g., in the “hallmarks of aging” [13]) are not sufficiently supported by studies that provide organismal-level aging data. As a consequence, it is not currently clear to what extent any of these putative aging regulators is in fact broadly linked to organismal aging rate. As outlined above, available data, in contrast, suggest that even interventions commonly claimed to “slow aging” in fact have little effect on most age-dependent phenotypic change. Future research can build on unbiased, multidimensional analyses of aging to determine the extent to which molecular regulators with a proposed role in aging do in fact (or do not) influence (aspects of) the aging process.

Aging research essentially deals with a many-to-many mapping problem. There are changes in many age-sensitive phenotypes (collectively representing the aging process, i.e., the transition of a young adult organism to an aged one) that could, in theory, each be influenced by a large set of regulators. Advances in aging research will critically depend on a better definition of this problem. Some important outstanding questions along those lines are:

- To what extent do aging-associated phenotypic changes cluster vs. are independent of each other?
- Did regulators of age-dependent phenotypic change evolve or do they not exist? It is clear that regulators for some aspects of age-dependent change did not evolve (e.g., neurons lost during aging do not get replaced; no mechanisms exist to repair certain changes of the extracellular matrix), but the answer will differ from phenotype to phenotype.
- What are possible regulators of age-dependent phenotypic change (including the putative aging regulators discussed here as well as others)? Again, this needs to be considered on a phenotype-by-phenotype basis.
- How complex are aging regulators in biological systems (e.g., how many factors may be required to act in concert to modify change of a phenotype)?
- More generally, how many regulators map onto change in how many phenotypes? The extreme ends of the spectrum of possible scenarios are marked by scenarios in which 1) each of a large number of age-dependent phenotypic changes is influenced by a completely different set of regulators and 2) all age-dependent phenotypic change is jointly influenced by a small set of regulators. The middle ground between these extremes is occupied by scenarios in which there are themes of regulators common to subsets of age-dependent phenotypic change.

In conclusion, aging research will benefit from a better definition of how specific regulators map onto age-dependent change, considered on a phenotype-by-phenotype basis. Resolving some of these key questions will shed more light on how tractable (or intractable) the biology of aging is.

## Supplementary information

Supplementary Table 1

Supplementary Table 2

Supplementary Table 3

Supplementary Table Legends
