# Supplementary material for: Targeting the “hallmarks of aging” to slow aging and treat age-related disease: fact or fiction?
Source: Mol Psychiatry. 2022 Jul 15;28(1):242–55. doi: 10.1038/s41380-022-01680-x (PMC9812785; doi:10.1038/s41380-022-01680-x)
Supplement: Supplementary file 2 — Supplementary Table 2 [file 41380_2022_1680_MOESM2_ESM.docx]

**Supplementary Table 2**

| **“Hallmarks of aging”** | **Name of the intervention (experimental condition)** | **Yeast** | **Worms** | **Flies** | **Mice** |
| --- | --- | --- | --- | --- | --- |
| **Genomic instability** | Overexpression BubR1 | - | - | - | Yes |
| **Telomere Attrition** | Tert | - | - | - | Yes |
| **Epigenetic alteration** | ASH-2 (deficiency) | - | Yes | - | - |
|  | PRC2  (mutation) | - | - | Yes (male) | - |
|  | Sir2 (overexpression) | Yes | Yes (but into question due to genetic background issue) | Yes (but into question due to genetic background issue) | - |
|  | Overexpressing Sirt6 | - | - | - | Yes (only in male but not female) |
|  | HP1α overexpression | - | - | Yes (only female) | - |
|  | Mir 71 | - | Yes | - | - |
|  | Upregulation of mir-31 | - | Yes (male) | - | - |
| **Loss of Proteostasis** | Hsp22 (overexpression) | - | - | Yes (male) | - |
|  | HSP-16 (overexpression) | - | Yes | - | - |
|  | HSF-1(activation) | - | Yes | - | - |
|  | Thioflavin T  (Amyloid-binding compounds) | - | Yes | - | - |
|  | Rapamycin | Yes | Yes | Yes (both male and female but the effect on the female is much bigger) | Yes |
|  | Spermidine | Yes | Yes | Yes (only female) | Yes |
|  | Dietary supplementation with ω-6 polyunsaturated fatty acids | - | Yes | - | - |
|  | *let-23* (gain-of-function) | - | Yes | - | - |
|  | Activation of Rpn4 | Yes | - | - | - |
|  | RPN-6 (overexpression) | - | Yes | - | - |
| **Deregulated Nutrient-sensing** | IIS pathway and growth hormone | Yes | Yes | Yes (mainly female, or much greater impact on female compared to male) | Yes |
|  | Pten-overexpressing | - | - | - | Yes |
|  | PI3K (partial loss) | - | - | - | Yes |
|  | mTORC1 (down-regulation) | Yes | Yes | Yes | Yes |
|  | S6K1 (deficient) | - | - | - | Yes |
|  | Metformin | - | Yes | - | Yes |
|  | Dietary restriction | Yes | Yes | Yes (mainly female, or much greater impact on female compared to male) | Yes |
| **Mitochondrial Dysfunction** | ROS activation | Yes | Yes | - | - |
|  | PGC-1α (overexpression in the intestine) | - | - | Yes (female) | - |
|  | Uncoupling protein UCP1 (overexpression) | - |  | Yes (male and female but much greater effect has been seen in females) | Yes |
| **Cellular Senescence** | Systematic increase in the expression of p16^Ink4a^ | - | - | - | Yes |
|  | Activation of P53 | - | - | - | Yes |
| **Stem Cell Exhaustion** | PGC-1 (overexpression) | - | - | Yes (only in females) | - |
| **Altered Intercellular Communication** | aspirin | - | - | - | Yes (in males) |
|  | Inhibition of NF- κB | - | - | - | Yes |
