# Supplementary material for: Targeting the “hallmarks of aging” to slow aging and treat age-related disease: fact or fiction?
Source: Mol Psychiatry. 2022 Jul 15;28(1):242–55. doi: 10.1038/s41380-022-01680-x (PMC9812785; doi:10.1038/s41380-022-01680-x)
Supplement: Supplementary file 4 — Supplementary Table Legends [file 41380_2022_1680_MOESM4_ESM.docx]

**Supplementary Table Legends**

**Supplementary Table 1**: We identified all the references which have been presented in the "hallmarks of aging" paper [13] as evidence for the causal involvement of each of the hallmarks in the aging process. We analyzed each paper based on the following criteria: 1) whether the effect (on lifespan and/or aging-associated change) has been shown in the context of natural aging, 2) whether a lifespan extension was shown, 3) whether anti-aging effects (i.e., a countering of aging-associated alterations by treatment) have been claimed (and, if yes, how many aging-associated parameters this claim was based on), 4) whether the age-sensitivity of parameters has been demonstrated in the paper (by showing a difference between young and old control animals), 5) whether young treated animals were included in the study design (and, if yes, whether treatment effects can also be seen in young animals or not).

**Supplementary Table 2**: The table summarizes all the genetic variants as well as dietary factors and pharmacological treatments which have been shown to induce lifespan extension according to studies cited in the “hallmarks of aging” paper [13]. This summary is restricted to studies focused on the analysis of natural aging.

**Supplementary Table 3**: An important set of interventions for the aging field represent those targeting (inhibiting) growth hormone signaling. We searched for relevant papers in PubMed using the following search term: (aging[Title/Abstract] OR ageing[Title/Abstract] OR healthspan[Title/Abstract]) AND (mice[Title/Abstract] OR mouse[Title/Abstract]) AND (Ghrhr[Title/Abstract] OR Ames[Title/Abstract] OR Prop1[Title/Abstract] OR Snell[Title/Abstract] OR Pit1[Title/Abstract] OR Laron[Title/Abstract] OR Ghr[Title/Abstract] OR "Igf1 heterozygous"[Title/Abstract] OR "Igf1 haploinsufficiency"[Title/Abstract]) NOT (REVIEW OR COMMENT OR COMMENTARY). At the time of the search (February 2022), this gave 178 results. The 178 papers were then further screened for relevancy. Reviews, commentaries and non-English papers were excluded, as well as papers where we did not have access to the full-text. In addition, papers were excluded for the following reasons: non-relevance (e.g. different PiT term), no mouse studies, or no aged animals. Despite their importance for aging studies, we did not include an analysis of omics datasets, since we did not have all the information necessary to fit them to the scope of Supplementary Table 3.
